# Multi-UAV Collaborative Target Search Method in Unknown Dynamic Environment

**DOI:** 10.3390/s24237639

**Published:** 2024-11-29

**Authors:** Liyuan Yang, Yongping Hao, Jiulong Xu, Meixuan Li

**Affiliations:** School of Equipment Engineering, Shenyang Ligong University, Shenyang 110159, China; 2100600007@stu.sylu.edu.cn (L.Y.); xujiulong@sylu.edu.cn (J.X.); 2200600015@stu.sylu.edu.cn (M.L.)

**Keywords:** multi-UAV cooperative search, dynamic objectives, local particle swarm optimization algorithms, adaptive planning

## Abstract

The challenge of search inefficiency arises when multiple UAV swarms conduct dynamic target area searches in unknown environments. The primary sources of this inefficiency are repeated searches in the target region and the dynamic motion of targets. To address this issue, we present the distributed adaptive real-time planning search (DAPSO) technique, which enhances the search efficiency for dynamic targets in uncertain mission situations. To minimize repeated searches, UAVs utilize localized communication for information exchange and dynamically update their situational awareness regarding the mission environment, facilitating collaborative exploration. To mitigate the effects of target mobility, we develop a dynamic mission planning method based on local particle swarm optimization, enabling UAVs to adjust their search trajectories in response to real-time environmental inputs. Finally, we propose a distance-based inter-vehicle collision avoidance strategy to ensure safety during multi-UAV cooperative searches. The experimental findings demonstrate that the proposed DAPSO method significantly outperforms other search strategies regarding the coverage and target detection rates.

## 1. Introduction

In fields such as marine biological monitoring and wilderness search and rescue, UAVs are often regarded as the optimal choice for performing dynamic target search tasks due to their advantages of zero personnel casualties, low production costs, and superior size, speed, and maneuverability [1,2]. With the increasing complexity of missions and environments, a single UAV is no longer sufficient to meet mission requirements. Consequently, UAV cooperative systems composed of multiple UAVs have garnered significant attention from researchers [3,4]. The cooperative execution of tasks by multiple UAVs not only expands the operational range, reduces the mission execution time, and improves task efficiency but also highlights the flexibility and robustness of multi-UAV cooperative systems [5]. Multi-UAV systems achieve the spatiotemporal collaboration of situational awareness information by enabling mutual communication and sharing local environmental information among the UAVs [6,7].

Driven by practical demands, research on multi-UAV cooperative target search methods builds upon collaborative technologies to leverage the performance advantages of multiple UAVs. Based on the existing theories of persistent target detection and searching, this research addresses real-world target search challenges, providing a practical platform for advancing theories and technologies in multi-UAV cooperation, computer vision [8], and aircraft design and manufacturing. However, conducting target search missions with multiple UAVs presents specific challenges. The limited sensing range of platforms, the complexity of mission environments, and the uncertainty of target information leads to low efficiency in task execution using existing methods and platforms. Therefore, designing an effective cooperative search method to ensure coordination among multiple UAVs is particularly critical [9].

In multi-UAV cooperative target search missions, targets are categorized into static and dynamic targets based on their motion characteristics. Static targets are ones whose position in the search area is fixed or changes so slowly that it can be approximated as static during the search. To search for static targets [10], earlier domestic and international studies were based on global communication conditions and transformed the static target search problem into a static area coverage problem. For different mission scenarios, researchers have proposed a variety of trajectory planning methods, including regional scanning based on zigzag formations [11], a parallel interval search strategy using Dobbins curves [12], and coverage algorithms such as the helix method and spanning tree algorithms for solving the problem. Since the global path is pre-planned, these methods, although capable of providing complete coverage of the task area without omissions, are limited when a UAV experiences failure or other unforeseen circumstances. Additionally, they do not account for the impact of UAV performance limitations, which can lead to longer search paths and, consequently, reduced search efficiency.

In recent years, the focus of domestic and international research has gradually shifted to moving targets and dynamic environments [13]. Due to the uncertainty of the target position and motion state when the target is in motion, there are still many problems in search missions for moving targets to be solved [14,15]. How to decrease the search duration and improve the search efficiency for moving targets are the core issues of the moving target search problem. Target probability maps [16,17] and pheromone-based mechanisms [18,19] have been widely used in moving target search problems. The former is primarily used to describe the target’s location information, while the latter simulates the release, propagation, and detection of pheromones to facilitate the search for moving targets. References [20,21] use probability functions to describe the target model, updating the target probability for each grid. The higher the probability, the greater the likelihood that the target exists. This approach effectively enables the search for unknown targets in complex environments. However, it does not account for the false alarm probability of the sensors. Collaborative multi-UAV search methods based on pheromone mechanisms can only be limited to smaller scale environments and are challenging in terms of practical applications and computational complexity. Biologically inspired algorithms, including genetic algorithms [22], ant colonies [23], and bionic algorithms such as bird flocks [24], have also been applied to search task planning. The literature [25] proposes the Motion-Coding Particle Swarm Optimization (MPSO) algorithm to achieve the optimal searching of UAVs for moving targets. Reference [26] presents a multi-UAV target search method based on the pigeon flock optimization algorithm, which is improved using a differential evolution strategy. This algorithm assumes that the target releases pheromones while moving. The above literature assumes that the target information is known. Reference [27] proposes a UAV cooperative search method for moving targets in unknown environments, where each UAV adopts two flight modes. In the cluster mode, a local particle swarm search model is dynamically constructed in real time, allowing more targets to be found in a shorter period. However, due to environmental uncertainty, the algorithm only considers the target’s existence probability, which presents a certain degree of bias [28,29].

It can be seen from the literature that although all the above methods can locate the moving targets, they rarely consider the uncertainty and unknown nature of target information at the initial time, as well as the impact of area coverage on search efficiency. Furthermore, most algorithms assume full communication connectivity for cooperation, which does not align with real-world scenarios. Therefore, we fully consider the UAVs’ level of environmental awareness. While planning search trajectories based solely on local exploration information, we also incorporate an inter-vehicle collision avoidance mechanism to improve the overall search efficiency and ensure the safety of the UAVs.

According to the literature, while all these methods can discover a moving target, they rarely account for the target’s unpredictability and require multiple scans of the area to detect it, limiting the overall search efficiency [30,31]. As a result, we investigate the UAV’s level of environmental awareness and arrange the search trajectory based solely on local exploration information [32,33]. When multiple UAVs perform search tasks for dynamic targets in uncertain mission environments, this paper proposes a distributed dynamic real-time planning search strategy based on local particle swarm optimization to ensure target search efficiency and quickly reduce environmental uncertainty. We assume that the target moves at a constant pace and that the direction of motion is random and does not cause significant variations. Each UAV in the multi-UAV system can plan its trajectory based on its current state, surroundings, and local information interactions. The primary contributions of our study are as follows:

- Due to the uncertainty and dynamic changes in the environment and search targets, the UAVs perform trajectory planning based on their state and surrounding environmental information. They establish a model of target probability distribution with uniform distribution, providing an essential prior probability distribution. The influence of the sensor detection probability and false alarm rate is also considered. The UAVs update their information based on local and collaborative interactions.
- To thoroughly assess the level of environmental uncertainty, we devised a TA function, an exploration gain function, and an attractiveness evaluation function. The objective function with dynamically changing parameters becomes known, and the UAV search trajectory is dynamically adjusted based on the current environmental information and search progress, transforming the search problem into a trajectory optimization problem. Finally, the DAPSO algorithm employs the UAV’s optimal search trajectory.
- When using the particle swarm optimization technique for the optimal search trajectory planning of UAVs, a distance-measuring inter-machine collision avoidance mechanism is devised to ensure UAV safety. During the search process, the UAV detects the distance to nearby UAVs in real time, and the collision avoidance system is activated if a potential collision risk becomes apparent.

The remainder of the paper appears as follows. Section 2 introduces the multi-unmanned vehicle cooperative target search issue and the corresponding mathematical model. Section 3 goes over the cooperative search objective function in depth. Section 4 outlines the trajectory updating approach used in the UAV search operation. Section 5 simulates the same scenario and compares the results to various methods. Finally, Section 6 summarizes the significant findings and suggests future study topics.

## 2. Related Work

**Figure 1:**
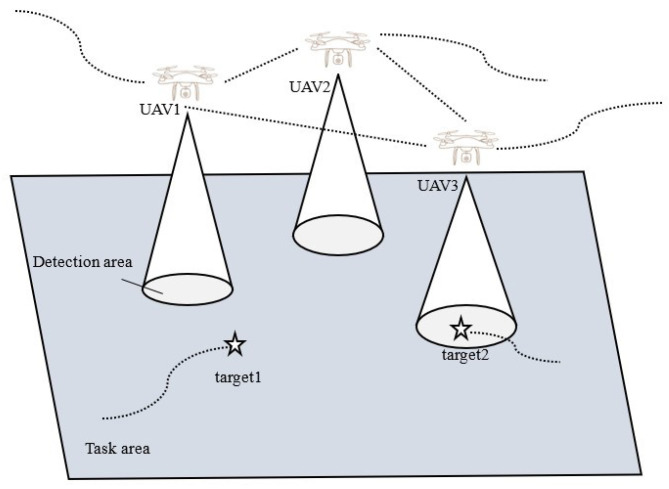
UAV coordinated target search scenario.

Figure 1 depicts a multi-UAV cooperative search for multiple moving targets in an uncertain mission area Ω. UAVs are denoted as $U_{i}$, where $i\in \left\{1,2,3,\ldots N\right\}$, and targets are denoted as $T_{i}$, where $i\in \left\{1,2,3,\ldots M\right\}$. The targets move at the same speed throughout the zone, with generally stochastic trajectories. Since the number and initial locations of the targets are unknown, each UAV is equipped with a downward-facing detection camera to search the region and estimate the number and positions of the targets. Assuming that all UAVs fly at the same altitude and speed, they exchange real-time data within their communication range.

### 2.1. UAV Motion Model

When a UAV executes a search mission, the UAV performs an isometric flight at a set speed. Because it only focuses on the drone’s trajectory, the UAV is treated as a moving mass in a two-dimensional space using the simplified equation of motion.

(1) $$\left\{\begin{array}{c} \dot{x}=v\cos\varphi \\ \dot{y}=v\sin\varphi \\ \dot{\varphi }=\omega \end{array}\right.$$

where $\left(x,y\right)\in R^{2}$ represents the UAV’s horizontal position in the mission area, *v* is its flying speed, and φ is its yaw angle.

### 2.2. Sensor Model

**Figure 2:**
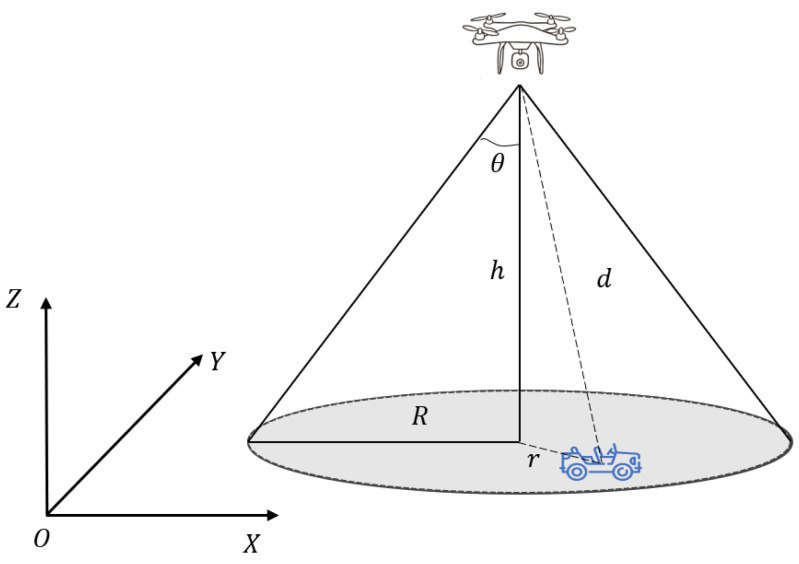
Schematic diagram of the sensor.

The UAV is equipped with a visual sensor to scan ground targets, as shown in Figure 2. Assuming that the visual sensor’s projection on the ground is circular, the detection range is proportional to the UAV’s flying height, and due to UAVs flying isometrically, their detection ranges are constant and consistent. In the diagram, h represents the UAV’s current flight height, R is the detection radius of the vision sensor, and the relationship between them is

(2) R=htanθ

The three-dimensional field-of-view search decreases to a two-dimensional planar scanning issue, with the target deemed to be detected if it occurs in the circular detection area.

### 2.3. Environmental Model

**Figure 3:**
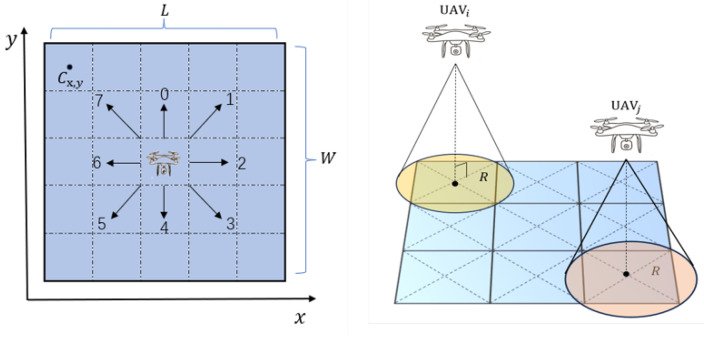
Task area rasterization.

To make it easier to describe the target search process and simplify the solution space for collaborative decision-making, the task area is rasterized to create the environment map shown in Figure 3. Assuming the job area’s length and breadth are *L* and *W*, respectively, the minimum grid should be divided by the maximum inner tangent rectangle of the sensor detection area to ensure that no area gets overlooked when the UAV scans the minimum grid cell. Because the square has the largest area in the tangent rectangle of a circle, the grid size is configured to a square with a side length of 2R. During the search, each UAV occupies one grid, and the viable domain consists of the eight grids nearest to itself.

Each cell is represented by $C_{x,y}$, where $\left(x,y\right)$ is the grid’s row and column coordinates, and each grid can only contain one target.

### 2.4. Target Probability and Updating Model

UAVs operating in an unfamiliar environment lack precise prior information on the target’s location when they undertake mobile target searches. The initial assumption is that there is an equal chance of the target being in each grid to keep UAVs from overly focusing on one area. The target is uniformly distributed over the mission area and described using a probability distribution function.

(3) $$P_{x,y}^{i}\left(t\right)=\frac{1}{n}$$

where *n* is the total number of grids and $P_{x,y}^{i}\in \left[0,1\right]$ is the target probability. As the search progresses and new data are acquired, the probability of the target being in each grid is updated based on the observation data.

As the UAV search progresses, updating the target probabilities is a critical step that directly affects the success rate of the search. Due to limitations from environmental noise and the sensor’s performance, there is an impact on the detection accuracy. Therefore, we considered both the sensor’s detection possibility and false alarm probability to enhance the model’s realism and practicality.

$O_{x,y}\left(t\right)$ represents the UAV observation result at time t for the position $\left(x,y\right)$ and $E_{x,y}\left(t\right)$ indicates whether the target is in the cell at that time. $P_{t}$ is the likelihood of sensor detection, while $P_{f}$ represents the rate of false alarms. Using the Bayesian updating criterion to update the target presence probability based on observed data, if the sensor identifies a target in grid $\left(x,y\right)$, the updating formula is

(4) $$P_{x,y}\left(t+1\right)=\frac{P_{t}P_{x,y}\left(t\right)}{P_{t}P_{x,y}\left(t\right)+P_{f}\left(1-P_{x,y}\left(t\right)\right)}\;O_{x,y\left(t\right)}=1$$

If the sensor does not detect a target in grid $\left(x,y\right)$, the updating formula is

(5) $$P_{x,y}\left(t+1\right)=\frac{\left(1-P_{t}\right)P_{x,y}\left(t\right)}{\left(1-P_{t}\right)P_{x,y}\left(t\right)+\left(1-P_{f}\right)\left(1-P_{x,y}\left(t\right)\right)}\;O_{x,y\left(t\right)}=0$$

## 3. Multi-UAV Collaborative Goal Search Optimization Function

### 3.1. Environmental Uncertainty Quantization Function

Information entropy mainly measures the degree of uncertainty in the system. Because the mission area is unknown at the initial moment, and the information of the moving target in each grid is uncertain, we use information entropy to quantify the uncertainty of the environment, and each UAV maintains a map of its uncertainty of the environment, and the information entropy is updated in real time according to the observation information obtained in the course of searching. Areas with a higher information entropy, which indicates significant uncertainty, direct the UAV’s search to improve coverage while lowering environmental uncertainty.

(6) $$H_{x,y}\left(t\right)=-\left(P_{x,y\left(t\right)}\log P_{x,y}\left(t\right)+\left(1-P_{x,y}\left(t\right)\right)\log\left(1-P_{x,y}\left(t\right)\right)\right)$$

$P_{x,y}\left(t\right)$ represents the probability that the target exists in the grid $\left(x,y\right)$.

### 3.2. Exploring the Gain Function

The exploration gain function is intended to quantify the potential value of the newly explored region relative to the explored region during the exploration process, use region coverage as an evaluation index of search efficiency, and optimize resource allocation, thereby guiding the search strategy. The task area is separated into n grids, with each grid represented by its center point coordinates $\left(x,y\right)$, where $x,y\in \left\{1,2,3,\ldots ,n\right\}$.

Assume each mesh $\left(x,y\right)$ has a covering state $C_{x,y}\left(t\right)$, designated as follows:

(7) $$C_{x,y}\left(t\right)=\left\{\begin{array}{c} 1,\;has\;been\;searched\\ 0,\;not\;searched \end{array}\right.$$

The area coverage is the proportion of the total mission area that the UAV has searched, and the coverage *R* may be defined as follows:

(8) $$R\left(t\right)=\frac{\sum_{x=1}^{n}\sum_{y=1}^{n}C_{x,y}\left(t\right)}{n^{2}}\times 100\%$$

The exploration gain from *t* to t+1 is the difference between $R\left(t+1\right)$ and $R\left(t\right)$:

(9) $$I\left(t\right)=R\left(t+1\right)-R\left(t\right)=\frac{\sum_{x=1}^{n}\sum_{y=1}^{n}C_{x,y}\left(t-1\right)-C_{x,y}\left(t\right)}{n^{2}}$$

The exploration gain function can efficiently avoid redundant searches in the same area, resulting in less work and resource loss.

### 3.3. Attractiveness Assessment Function

When a UAV performs a target search mission, the number of visits to a grid is a vital indicator due to the rasterization of the mission area, so establishing the grid attractiveness evaluation function and focusing on this metric later in a multi-UAV collaborative target search mission can improve the target detection probability.

$V_{x,y}^{i,t}$ represents the total number of searches in cell $C_{x,y}$ by all UAVs until the moment t. UAVs must add one after each search.

(10) $$V_{x,y}\left(t\right)=\left\{V_{x,y}^{i,t},C_{x,y}\in \Omega \right\}$$

So, the attractiveness evaluation function is as follows:

(11) $$A_{x,y}\left(t\right)=\frac{1}{V_{x,y}\left(t\right)+1}$$

Areas with fewer visits should be more attractive to drones, and our approach gives them a higher priority.

## 4. Distributed Collaborative Search Methods for Uncertain Environments

### 4.1. Distributed Search Decision Framework for Multi-UAV Swarms

**Figure 4:**
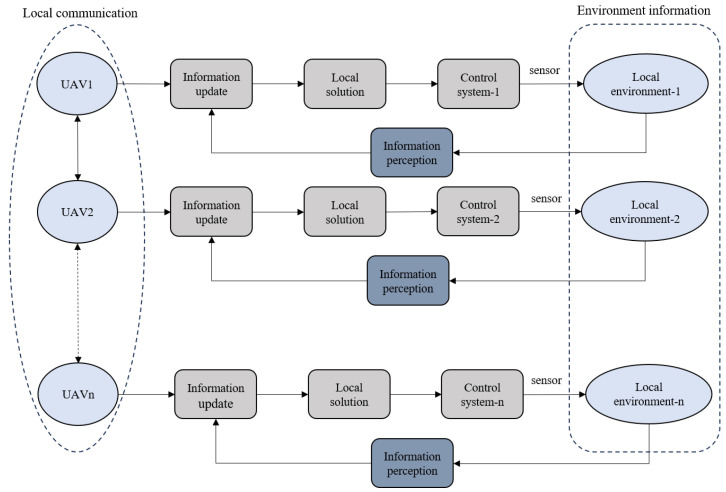
Multi-UAV cooperative search framework.

The multi-UAV collaborative search work uses distributed control, and the UAV possesses monadic intelligence, which reduces a single UAV’s processing capacity through data sharing while having no influence on the overall task when a UAV fails to accomplish the mission. The UAV’s initial position is random, and each UAV begins the search task from there. Each UAV scans the task area with its onboard camera, fuses and updates the local target information it identifies with the self-information, and makes an autonomous decision about its flight direction, with single-unit decision-making being generally independent. The UAV contains a communication device that allows it to engage with neighboring UAVs within its communication range to update their local information, calculate the next moment position information using algorithmic solutions, and regulate the UAV’s flight route. The multi-UAV cooperative search framework is in Figure 4 below.

### 4.2. Adaptive Trajectory Planning Based on Localized Particle Swarms

When multiple UAVs search for moving targets in a coordinated manner, the dynamic environment, uncertainty of the target, and sensor limitations increase the complexity of the search. The critical problem is how to coordinate the UAVs to improve the target detection probability while rapidly reducing environmental uncertainty. Particle swarm optimization algorithms show significant advantages in solving the above problems. The PSO algorithm guides the search process by simulating the hunting behavior of a flock of birds, using the information shared by the individuals in the flock. Each UAV can be regarded as a particle that updates its flight path by tracking the best positions encountered by the two “extreme” individuals and the best positions encountered by all particles in the flock. This approach allows the PSO algorithm to efficiently explore the solution space while retaining the pursuit of a globally optimal solution.

(12) $$v_{i}\left(t+1\right)=\omega \cdot v_{i}\left(t\right)+c_{1}\cdot r_{1}\cdot \left(pbest_{i}-x_{i}\left(t\right)\right)+c_{2}\cdot r_{2}\cdot \left(gbest_{i}-x_{i}\left(t\right)\right)$$

(13) $$x_{i}\left(t+1\right)=x_{i}\left(t\right)+v_{i}\left(t+1\right)$$

ω is the inertia weight, which controls the breadth and depth of the search, $c_{1}$ is the self-awareness coefficient, $c_{2}$ is the social learning coefficient, and $r_{1}$ and $r_{2}$ are random numbers between $\left[0,1\right]$, which increase the randomness and diversity of the particles’ movement.

The particle swarm algorithm assumes that the particles synchronize their information after each iteration and that all particles have instant access to the global best position of the entire population, regardless of their position in the search space. However, this is contrary to the actual environmental situation, where the communication range between UAV individuals is limited, decision-making is only dependent on the information of the surrounding neighbors, and the environmental information held by different UAV individuals is inconsistent. Therefore, we propose a multi-UAV distributed dynamic real-time planning search method (DAPSO) based on local particle swarm optimization.

When multiple UAVs perform search tasks, they cooperate to complete the detection and localization of the target area. This method can dynamically adjust the search strategy according to environmental changes and task requirements, enhancing the flexibility and adaptability of the search task. Through distributed cooperation, even if some UAVs experience failures or communication interruptions, other UAVs can continue the search task, ensuring the system’s robustness.

Let $f\left(C_{x,y}\left(t\right)\right)$ be the fitness function, defined as follows:

(14) $$f\left(C_{x,y}\left(t\right)\right)=\omega_{1}H_{x,y}\left(t\right)+\omega_{2}I\left(t\right)+\omega_{3}A_{x,y}\left(t\right)$$

where $\omega_{1},\omega_{2},\omega_{3}$ are the weighting coefficients $\omega_{1}+\omega_{2}+\omega_{3}=1$, greater environmental uncertainty, a greater probability of the target’s existence, and a greater value of the fitness function. Therefore, the main focus is to consider the exploration gain and environmental uncertainty in the early search stages and the attractiveness of the grid in the later stages of the search.

In two-dimensional space, the best position is when the fitness is maximal at a given moment *t*:

(15) $$f\left(C_{x,y}^{i,l}\left(t\right)\right)=max\left\{f\left(C_{x,y}^{i}\left(t\right)\right)\right\}$$

Furthermore, once the distance between particles approaches the communication range for information exchange, updating the global optimal position depends on the local optimal position stated in Formula (16) to obtain

(16) $$\Delta_{x,y}\left(t\right)=\left\{f\left(C_{x,y}^{i}\left(t\right)\right)\geq f\left(C_{x,y}^{j}\left(t\right)\right)\right\}$$

In each iteration cycle, the speed and position of the UAV updates as follows:

(17) $$V_{x,y}^{i}\left(t\right)=\omega V_{x,y}^{i}\left(t-\tau \right)+c_{1}r_{1}V_{x,y}^{i,l}\left(t-\tau \right)+c_{2}r_{2}V_{x,y}^{i,g}\left(t-\tau \right)$$

(18) $$V_{x,y}^{i,l}\left(t-\tau \right)=\frac{C_{x,y}^{i,l}\left(t-\tau \right)-C_{x,y}^{i}\left(t-\tau \right)}{\tau }$$

(19) $$V_{x,y}^{i,g}\left(t-\tau \right)=\frac{\Delta_{x,y}\left(t-\tau \right)-C_{x,y}^{i}\left(t-\tau \right)}{\tau }$$

(20) $$C_{x,y}^{i}\left(t\right)=C_{x,y}^{i}\left(t-\tau \right)+V_{x,y}^{i}\left(t\right)\tau$$

### 4.3. Inter-Machine Collision Avoidance Strategy Based on Distance Measurement

**Figure 5:**
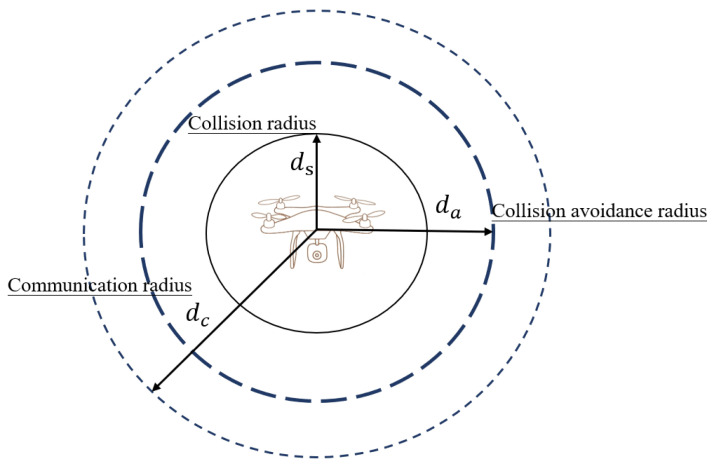
Measuring distance.

When UAVs performed cooperative search tasks, we designed a collision avoidance mechanism based on real-time distance detection in order to ensure effective communication and collision avoidance. During the search process, UAVs move toward the unsearched area based on their localized information, and each UAV detects the distance to other UAVs in real time and triggers information interaction if the distance between them reaches the communication range. Whenever the distance between two UAVs is less than $d_{c}$, the communication will be reliable. As shown in Figure 5.

(21) $$d_{ij}=\sqrt{{\left(x_{i}-x_{j}\right)}^{2}+{\left(y_{i}-y_{j}\right)}^{2}}$$

When the distance between UAVs, $d_{s}\leq d_{ij}\leq d_{a}$, is detected as a potential collision risk, the collision avoidance mechanism is activated. This mechanism adjusts the UAV’s flight speed and direction to avoid collisions while minimizing the impact on the search mission. Priority levels are assigned to the UAVs conducting the mission, and lower priority UAVs perform speed adjustments and evenly shift to the left side of the flying direction, assuring the overall safety and reliability of the UAV fleet during the mission. The speed adjustment formula is as follows:

(22) $$V_{x,y}^{i}\left(t\right)=V_{x,y}^{i}\left(t-1\right)+\sigma \frac{C_{x,y}^{i}\left(t-1\right)-C_{x,y}^{j}\left(t-1\right)}{d_{ij}}$$

σ is the collision avoidance adjustment factor, which is proportional to the distance between the UAVs.

## 5. Simulation Results and Analysis

To verify the feasibility and effectiveness of the algorithm proposed in this paper, we conducted simulation experiments and compared the simulation results with other algorithms, including a greedy search algorithm [21], random search algorithm, ant colony algorithm [34], and particle swarm algorithm [28], setting the parameters of the simulation experiment as follows: The search area is 30×30 cells with a square grid with a side length of 2 m. Five UAVs enter the environment at a random location in the mission area to search, and three moving targets have random positions in the environment; the radius of detection of the UAV’s sensor is 2 m. The detection probability is $P_{t}=0.8$, the false alarm rate is $P_{f}=0.2$, and the UAV’s radius of collision avoidance is $d_{a}$ = 2 m. Since the initial positions of the UAV and the moving target are random, and to eliminate chance introduced by the random factors, the simulation results are the average of several simulation experiments.

### Algorithm Metrics Comparison and Performance Analysis

**Figure 6:**
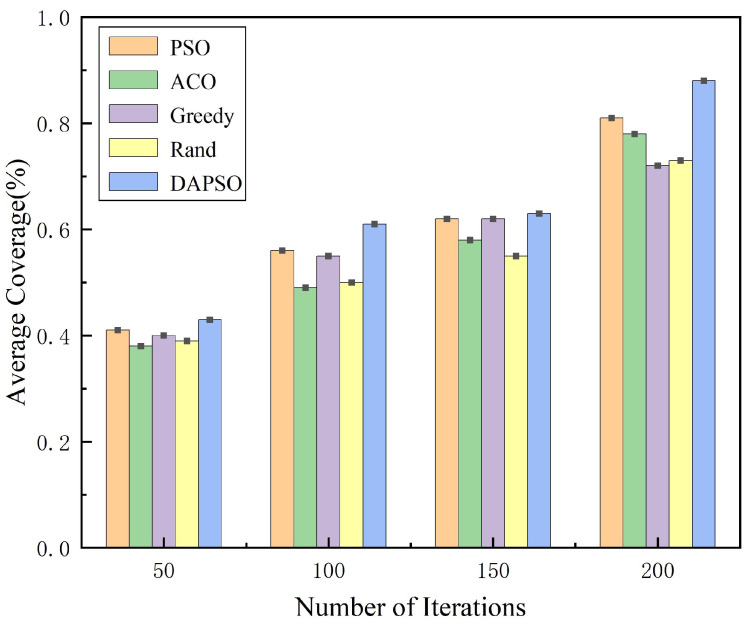
Histogram of the average coverage of the five search algorithms.

For the search for moving targets, the average area coverage was used as a performance index for evaluating the collaborative search algorithm, comparing the algorithm proposed in this paper with other search algorithms, and the data comparison of the simulation experiment results is shown in Figure 6 and Table 1. The search algorithms improved their coverage with the increase in the number of iterations; the DAPSO search algorithm of this paper has significantly higher coverage than the other algorithms in each iteration cycle, and the average coverage rate is close to 90% or so, indicating that the algorithm can effectively optimize its search path in each iteration, continuously expanding the coverage and avoiding the waste of resources caused by overlapping paths.

Because the DAPSO algorithm is an upgrade over the regular PSO algorithm, the search efficiency and accuracy are significantly higher. Although the coverage of the other algorithms also increases with the number of iterations, the growth is relatively small. This may be because the search strategies of these algorithms are relatively simple and fail to make full use of the feedback information in the iterations to optimize the search process. The effectiveness and convergence of the DAPSO search algorithm were verified to maximize the UAV search efficiency.

**Figure 7:**
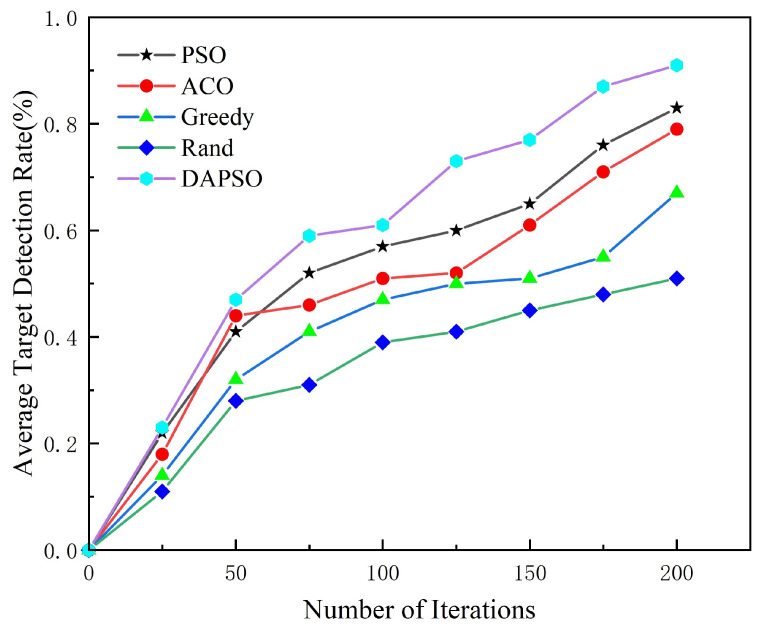
Average target detection rate.

Figure 7 shows the average target detection rate of five different algorithms with different iterations. The algorithms in the figure include the PSO, ACO, greedy, Rand, and DAPSO (improved particle swarm optimization algorithm) algorithms. From the figure, we can see that the average target search rate does not differ much in a short period, and with the search task continuing, the DAPSO algorithm proposed in this paper has the best average target discovery probability and a higher target search efficiency compared to the other search algorithms. The PSO and ACO algorithms perform well but are slightly inferior to DAPSO, showing the power of particle swarm and ant colony optimization algorithms in a target search. The greedy algorithm has some advantages at the initial stage but tends to fall into local optimums in long-term searching, though it led to a slow growth of the detection rate. The Rand algorithm performs the worst, which shows that it is difficult for a random search to find the target effectively without guidance. Therefore, the effectiveness of the proposed algorithm was verified.

**Figure 8:**
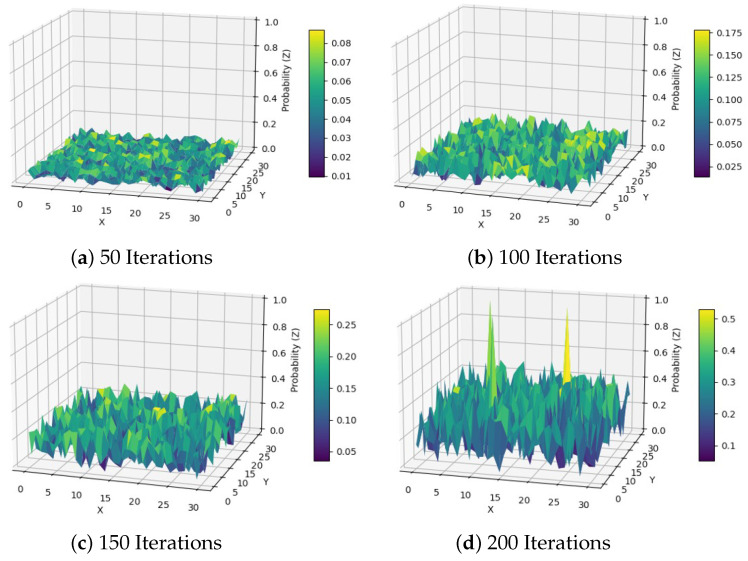
Target Detection Probability Convergence Plot.

Figure 8 demonstrates the variation in the target detection probability of the DAPSO algorithm with the number of search iterations under different iterations. It can observed from the figure that the target detection probability shows a gradual increase as the search process advances. In the initial stages of the algorithm, the target detection probability is relatively low due to the limited coverage of the search area. With the increase in the number of iterations, the UAV swarm gradually expands the search coverage through the adaptive adjustment of the DAPSO algorithm and improving the detection capability of the target area. This process reflects the adaptability and flexibility of the DAPSO algorithm in a dynamic environment. At the later stages of the algorithm iteration, the target detection probability stabilizes and reaches a high level, indicating that the DAPSO algorithm successfully locks the target. The experimental results verify the effectiveness of the DAPSO algorithm in a multi-UAV cooperative target search task.

**Figure 9:**
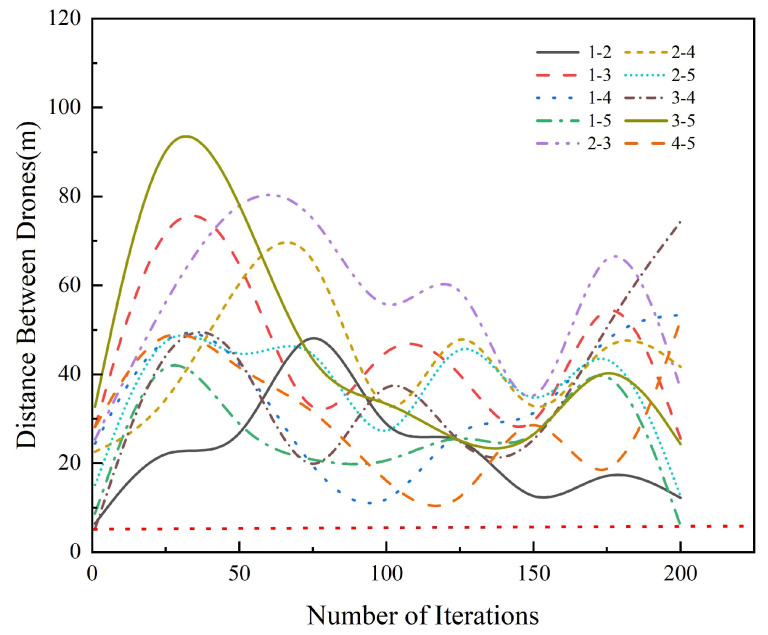
Drone–to–drone distance.

The inter-aircraft collision avoidance strategy based on distance measurement was added to the DAPSO algorithm to ensure the safety of UAVs when performing search tasks. The simulation results in Figure 9 show the change in distance between UAVs and the change in relative position during the search process through different colored lines, and it can be seen from the data that the distance between UAVs always kept above the safety threshold of 5 m, which verifies the effectiveness of the collision avoidance strategy. The strategy prevents potential collisions but has a negligible impact on the search efficiency, thus ensuring the safe and efficient execution of the search task.

## 6. Conclusions

This work proposes a multi-UAV distributed adaptive real-time planning search method (DAPSO) based on local particle swarm optimization to solve the challenge of a multi-UAV cooperative search for moving targets. Several simulation experiments were carried out and compared to other algorithms to ensure the usefulness of the suggested approach. The experimental results reveal that the DAPSO algorithm considerably increases the UAV swarm’s cooperative efficiency in the target search process while effectively reducing blindness. Compared to other traditional methods, the DAPSO algorithm demonstrates higher performance in the target discovery rate and area coverage. This proves the advantages of the DAPSO algorithm in enhancing the search efficiency but reflects its potential for adaptability to dynamic environments. Moreover, the collision avoidance strategy ensures the safety of drones during search missions, preventing potential collision risks. The algorithms operate in the most ideal communication environments, and we will further research the communication’s dependability.

## Figures and Tables

**Table 1:** Average coverage (%) for different iterations.

Algorithm	Iterations (Times)	Average Coverage (%)
PSO	50	0.41
	100	0.57
	150	0.65
	200	0.83
ACO	50	0.44
	100	0.51
	150	0.61
	200	0.79
Greedy	50	0.32
	100	0.47
	150	0.51
	200	0.67
Rand	50	0.28
	100	0.39
	150	0.45
	200	0.51
DAPSO	50	0.47
	100	0.61
	150	0.77
	200	0.91

## Data Availability

Data are contained within the article.
